# Glu_333_ in rabies virus glycoprotein is involved in virus attenuation through astrocyte infection and interferon responses

**DOI:** 10.1016/j.isci.2022.104122

**Published:** 2022-03-22

**Authors:** Yukari Itakura, Koshiro Tabata, Kohei Morimoto, Naoto Ito, Herman M. Chambaro, Ryota Eguchi, Ken-ichi Otsuguro, William W. Hall, Yasuko Orba, Hirofumi Sawa, Michihito Sasaki

**Affiliations:** 1 Division of Molecular Pathobiology, International Institute for Zoonosis Control, Hokkaido University, Sapporo, Hokkaido 001-0020, Japan; 2 Laboratory of Pharmacology, Department of Basic Veterinary Sciences, Faculty of Veterinary Medicine, Hokkaido University, Sapporo, Hokkaido 060-0818, Japan; 3 Laboratory of Zoonotic Diseases, Faculty of Applied Biological Sciences, Gifu University, Gifu, Gifu 501-1193, Japan; 4 National Virus Reference Laboratory, School of Medicine, University College of Dublin, Dublin 4, Ireland; 5 International Collaboration Unit, International Institute for Zoonosis Control, Hokkaido University, Sapporo, Hokkaido 001-0020, Japan; 6 Global Virus Network, Baltimore, MD 21201, USA; 7 One Health Research Center, Hokkaido University, Sapporo, Hokkaido 001-0020, Japan

**Keywords:** Immunology, Cellular neuroscience, Virology

## Abstract

The amino acid residue at position 333 of the rabies virus (RABV) glycoprotein (G333) is a major determinant of RABV pathogenicity. Virulent RABV strains possess Arg_333_, whereas the attenuated strain HEP-Flury (HEP) possesses Glu_333_. To investigate the potential attenuation mechanism dependent on a single amino acid at G333, comparative analysis was performed between HEP and HEP^333^R mutant with Arg_333_. We examined their respective tropism for astrocytes and the subsequent immune responses in astrocytes. Virus replication and subsequent interferon (IFN) responses in astrocytes infected with HEP were increased compared with HEP^333^R both *in vitro* and *in vivo*. Furthermore, involvement of IFN in the avirulency of HEP was demonstrated in IFN-receptor knockout mice. These results indicate that Glu_333_ contributes to RABV attenuation by determining the ability of the virus to infect astrocytes and stimulate subsequent IFN responses.

## Highlights

• Glu_333_ in G protein is responsible for astrocyte infection with RABV HEP strain
• Arg_333_ mutation in G protein decreases astrocyte tropism of RABV HEP
• RABV HEP evokes higher IFN responses in astrocytes than HEP with Arg_333_ mutation
• Glu_333_-dependent astrocyte infection is involved in the attenuation of RABV HEP

## Graphical abstract

**Figure:**
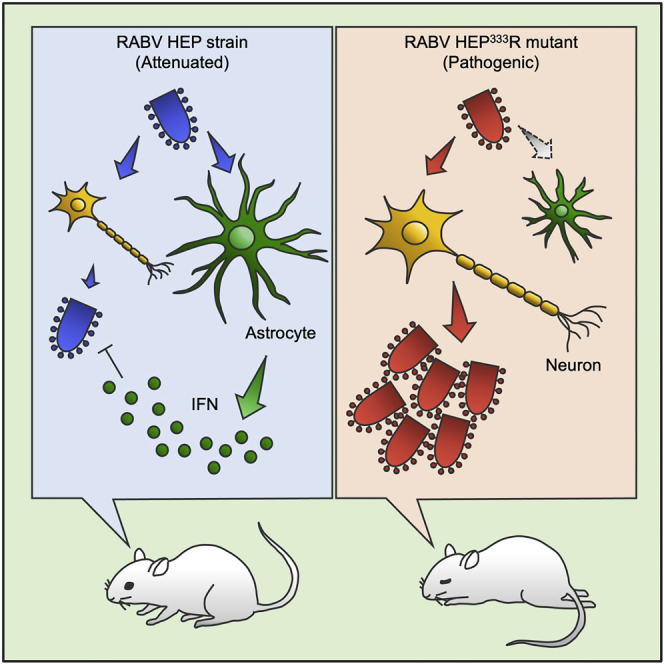

## Introduction

Rabies virus (RABV) is the causative agent of rabies, a fatal neurological disease in mammals causing at least 59,000 human deaths annually, particularly in Asian and African countries. Because of its global impact, rabies is notifiable to the World Health Organization (WHO) and International Epizootic Office (OIE) (Hampson et al., 2015; Singh et al., 2017). The RABV virion carries a negative-sense single-stranded RNA genome possessing five open reading frames encoding the nucleoprotein (N), phosphoprotein (P), matrix protein (M), glycoprotein (G), and large protein (L) (Finke and Conzelmann, 2005).

Highly pathogenic strains of RABV often exhibit characteristics related to their strict neurotropism (Ito et al., 2001; Morimoto et al., 1999, 2000). In contrast, attenuated strains often exhibit a broader cell tropism not specific to neuronal cells *in vitro* and show limited ability to spread to the central nervous system *in vivo* (Takayama-Ito et al., 2006; Tao et al., 2010). Among the viral proteins, the G protein exists as a trimer on the surface of the RABV virion. Because G protein is responsible for host cell receptor recognition (Sasaki et al., 2018; Thoulouze et al., 1998; Tuffereau et al., 1998; Wang et al., 2018) and membrane fusion (Gaudin et al., 1991), mutations in the G protein often alter viral pathogenicity. In particular, Arg at position 333 in the G protein (G333) contributes to virulence in some fixed strains of RABV in adult mice (Dietzschold et al., 1985; Ito et al., 2021; Morimoto et al., 2001; Nakagawa et al., 2012; Shuai et al., 2015). To understand this property, several studies have attempted to characterize the role of the amino acid at G333. An amino acid substitution for Glu_333_ enhanced viral-induced apoptosis in infected cells, leading to a loss of pathogenicity in mice (Tao et al., 2010). Infection with RABV encoding dual G proteins with Glu_333_ also resulted in enhanced apoptosis in cells (Faber et al., 2002). One study has reported that amino acid at G333 influences the binding affinity of G protein to one of the receptors for RABV, p75NTR, suggesting that amino acid change at G333 may affect the cell tropism of RABV (Tuffereau et al., 1998). Collectively, it has been clearly demonstrated that replacement of Arg_333_ or Lys_333_ to other amino acids causes a pathogenic shift of RABVs to an avirulent phenotype, whereas the mechanisms regulated by the amino acid residue at G333 remain unclear and controversial.

RABV infection of astrocytes has been reported to be dependent on the viral strain (Potratz et al., 2020). HEP-Flury, an attenuated RABV strain, shows low specificity in terms of the cell types it infects *in vitro* (Takayama-Ito et al., 2006), but shows high affinity for astrocytes *in vivo* (Mori and Morimoto, 2014). However, involvement of the amino acid residue at G333 in astrocyte infection is yet to be established. Recently, astrocytes abortively infected with diverse neurotropic viruses, including RABV, Theiler’s murine encephalomyelitis virus, and vesicular stomatitis virus, have been reported to be the main source of interferon (IFN)-β production in the brain conferring antiviral protection (Pfefferkorn et al., 2016). In addition, other studies showed that type-I IFN signaling in astrocytes is important to build an antiviral state in a virus-infected brain (Detje et al., 2015; Hwang and Bergmann, 2018; Kallfass et al., 2012; Pfefferkorn et al., 2016; Tian et al., 2018). RABVs are sensitive to IFN, and the importance of IFN in controlling RABVs has long been proposed (Postic and Fenje, 1971; Weinmann et al., 1979).

To further understand the attenuation mechanism dependent on the amino acid residue at G333 on aspects of viral infection and IFN responses in astrocytes, we investigated the tropism for astrocytes using a recombinant HEP-Flury strain (rHEP; Glu_333_) and a single amino acid mutant HEP^333^R strain (Arg_333_) *in vitro*. Infection of astrocytes and IFN responses were also examined *in vivo*. Finally, we examined the pathogenicity of rHEP in mice deficient in IFN signaling pathways, which might play a significant role in G333-dependent attenuation. Understanding the mechanism of RABV attenuation is essential in considering the use of live attenuated vaccines to control rabies in wild animals. Therefore, our present study has yielded new insights into the pathogenicity of RABV associated with G333.

## Results

### Impact of amino acid substitution at position 333 of the G protein on rRABV growth in neuron- and astrocyte-derived cell lines

**Figure 1:**
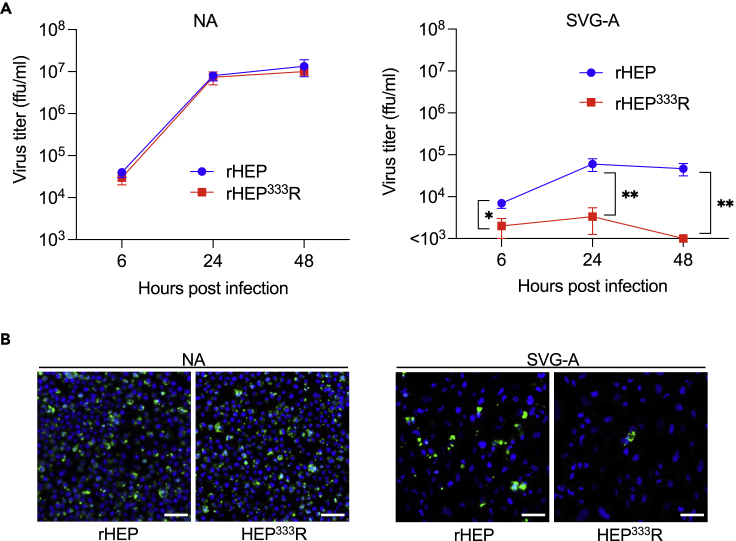
Infectivity of rabies virus (RABV) in neuron-derived NA cells and astrocyte-derived SVG-A cells Monolayers of NA or SVG-A cells were inoculated with rHEP or rHEP^333^R at a multiplicity of infection (MOI) of 1. (A) Viral growth curve. Supernatants were collected at the indicated time points, and virus titers were measured by a focus forming assay. Means ± standard deviations of triplicate data from a representative experiment are shown in the graph. A multiple t test was performed by the Holm–Sidak method for statistical analysis. ∗p < 0.05, ∗∗p < 0.01. (B) Images of RABV-infected cells. Cells were fixed at 24 h postinfection (hpi) and stained with FITC-conjugated anti-RABV N antibody for RABV N (green) and Hoechst 33342 for the nucleus (blue). The representative images were captured by fluorescent microscopic analysis. Scale bar, 50 μm.

To investigate the role of the amino acid at position 333 of RABV G protein (G333) on RABV infection, we generated recombinant RABV clones (rRABV) that included rHEP carrying Glu_333_ and rHEP^333^R carrying Arg_333_ in G protein by reverse genetics methods. First, we evaluated the replication efficacy between rHEP and rHEP^333^R in a neuron-derived cell line, NA cells, and an astrocyte-derived cell line, SVG-A cells. rHEP and rHEP^333^R showed similar growth kinetics in NA cells (Figure 1A). By contrast, in SVG-A cells, rHEP exhibited a significantly higher infectious virus titer at 24- and 48-h postinfection (hpi) compared with rHEP^333^R. No growth of rHEP^333^R was observed in SVG-A cells (Figure 1A). These results were reflected in the population of infected cells stained by FITC-labeled anti-RABV N antibody. In short, rHEP and rHEP^333^R exhibited similar infectivity to neuron-derived NA cells, whereas rHEP^333^R showed limited growth in astrocyte-derived SVG-A cells (Figure 1B). These results demonstrated that the amino acid residue at G333 influences the cell tropism of RABV.

### IFN production in primary astrocytes infected with rRABVs

**Figure 2:**
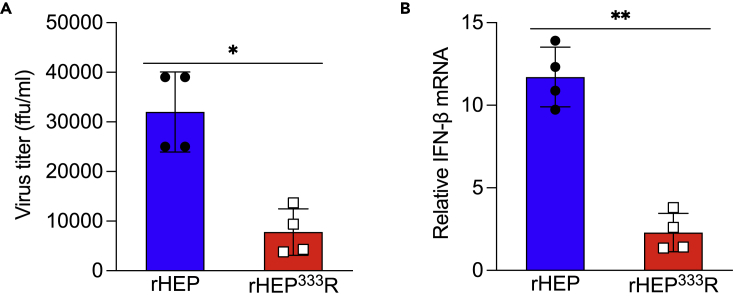
Infectivity of RABV in mouse-derived primary astrocytes (A and B) Astrocyte-derived primary cells were cultured on 24-well plates to 80% confluency and infected with rHEP or rHEP^333^R at an MOI of 1. Supernatants and RNAs were collected at 48 hpi and subjected to virus titration by a focus forming assay (A) and qRT-PCR for the measurement of IFN-β gene expression (B), respectively. Expression levels of the IFN-β gene were normalized to the β-actin gene and presented as fold changes relative to the mock controls using the ΔΔCt method. The values in the graph show the means ± standard deviations of a representative experiment. Statistical analysis was performed by the Student’s t test (∗p < 0.05, ∗∗p < 0.01).

Astrocytes act as IFN producers in brain tissue infected with neurotropic viruses (Detje et al., 2015; Hwang and Bergmann, 2018; Kallfass et al., 2012; Pfefferkorn et al., 2016; Tian et al., 2018), and thus we assessed the relationship between RABV infection and IFN responses in astrocytes. Primary astrocytes were used in these experiments because the astrocyte-derived cell line SVG-A lacks an IFN response against rRABV infection (Figure S1). In line with the results of rRABV growth in SVG-A cells (Figure 1), mouse-derived primary astrocytes were susceptible to rHEP infection but less susceptible to rHEP^333^R infection (Figure 2A). To confirm whether rRABV infection triggers IFN production in astrocytes, the mRNA levels of IFN-β in rRABV-infected primary astrocytes were quantified by qRT-PCR. The IFN-β gene expression levels in primary astrocytes were significantly higher in rHEP-infected cells than in rHEP^333^R-infected cells (Figure 2B). These results suggested that rHEP can infect astrocytes more efficiently and induce subsequent IFN production as compared with rHEP^333^R.

### rRABV tropism for astrocytes and the IFN responses *in vivo*

**Figure 3:**
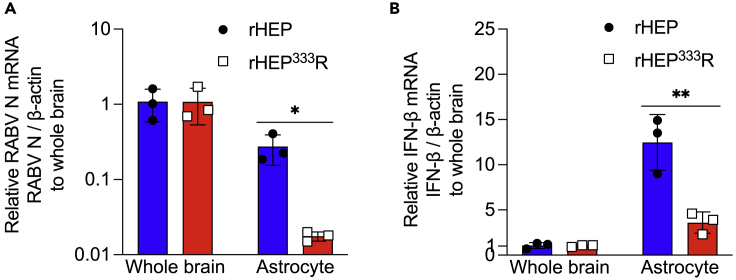
Infectivity of RABV and IFN expression in astrocytes in the brains of mice infected with rRABVs S129 mice were inoculated intracranially with 10^4^ focus forming units (ffu) of rHEP or rHEP^333^R. qRT-PCR was performed using homogenate of the brain tissue and isolated astrocytes at 5 days postinfection (dpi). (A) Relative RABV N mRNA level in astrocytes. The data were normalized to the β-actin gene and presented as fold changes relative to the whole brain using the ΔΔCt method. (B) Relative IFN-β mRNA expression in astrocytes. The data were normalized to the β-actin gene and presented as fold changes relative to the whole brain using the ΔΔCt method. All values in the bar graph show the means ± standard deviations of three mice from a representative experiment. Statistical analysis was performed by the Student’s t test (∗p < 0.05, ∗∗p < 0.01).

To further characterize the role of the amino acid at G333 with respect to astrocyte infection and IFN induction, we isolated astrocytes from the brains of immunocompetent S129 mice at 5 days after intracranial inoculation of rHEP or rHEP^333^R. To estimate the incidence of rRABV infection in astrocytes in the whole brain, we quantified viral RNA levels in astrocytes sorted from the rRABV-infected brain, and the data were described relative to viral replication in the whole brain. In rHEP-infected mice, viral RNA levels in astrocytes relative to the whole brain were significantly higher than these in rHEP^333^R-infected mice (Figure 3A), suggesting preferential infection of astrocytes by rHEP *in vivo*. Similarly, and consistent with the viral RNA levels, the gene expression levels of IFN-β were higher in astrocytes derived from rHEP-infected mice compared with those from rHEP^333^R-infected mice (Figure 3B). These results indicated that rHEP has tropism for astrocytes and induces high levels of IFN production in astrocytes *in vivo*.

### Comparison of infectivity and pathogenicity between rHEP and rHEP^333^R in IFN-receptor knockout (KO) mice

**Figure 4:**
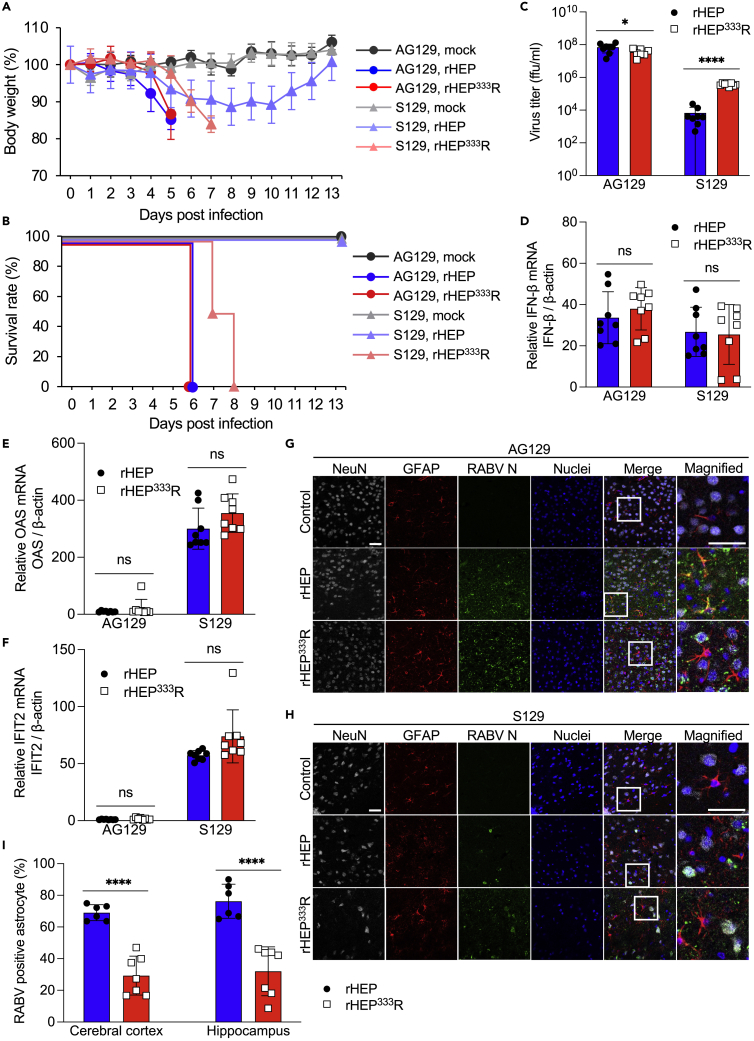
Evaluation of RABV pathogenicity in IFN-receptor knockout mice (A and B) Twelve-week-old AG129 or S129 mice were intracranially inoculated with 10^4^ ffu of rHEP or rHEP^333^R. Virus-infected mice were monitored for (A) body weight changes and (B) survival every day until 13 dpi. The values in the graph are shown as the means ± standard deviations (mock group; n = 4, virus challenge group; n = 8). (C) Virus titer in the 10% homogenate of the whole brain at 5 dpi was determined by a focus forming assay. The bar graphs show the means ± standard deviations (n = 8). (D–F) mRNA expression of IFN-β, OAS, or IFIT2 in the mouse brains at 5 dpi was determined by qRT-PCR. The results were normalized to the β-actin gene and presented as fold changes relative to the mock controls using the ΔΔCt method. The bar graphs show the means ± standard deviations (n = 8). Statistical analysis was performed by the Student’s t test (∗p < 0.05, ∗∗∗∗p < 0.0001). (G and H) Brain sections of (G) AG129 and (H) S129 mice at 5 dpi were stained for NeuN, GFAP, and RABV N protein. Scale bar; 20 μm. (I) RABV positive rate of astrocytes in AG129 mice. The number of RABV N-positive astrocytes were manually counted in 3 snapshots of cerebral cortex and hippocampus per section. Two animals were analyzed for each virus. Each dot represents positive rate of each snapshot. The bar graphs show the means ± standard deviations. Statistical analysis was performed by the Student’s t test (∗∗∗∗p < 0.0001).

rHEP is an attenuated RABV strain that causes transient infection in immunocompetent mice (Takayama-Ito et al., 2006). To evaluate the protective efficacy of IFN in mice infected with rHEP, we next examined the pathogenicity of rRABV in AG129 mice: type-I/II IFN receptor-knockout (KO) mice in an S129 background, which are highly susceptible to infection by various viruses (Aliota et al., 2016; Milligan et al., 2017; Tan et al., 2010). AG129 and S129 mice were infected with rRABVs intracranially to evaluate virus replication and host IFN responses in the central nervous system. Consistent with a previous report (Takayama-Ito et al., 2006), rHEP caused a transient decrease in body weight without any neurological symptoms in immunocompetent S129 mice (Figure 4A); however, rHEP^333^R showed body weight loss and neuropathogenicity at 6 days postinfection (dpi), resulting in 100% mortality by 8 dpi (Figure 4B). In contrast, in AG129 mice, rHEP as well as rHEP^333^R caused significant body weight loss and fatal neurological manifestations at 4–5 dpi, resulting in a 100% fatality rate at 6 dpi (Figures 4A and 4B). Furthermore, virus titration of the whole brain tissue at 5 dpi showed a high virus titer in S129 mice infected with rHEP^333^R compared with rHEP (Figure 4C). Conversely, there was no remarkable difference in virus titer in AG129 mice infected with rHEP or rHEP^333^R (Figure 4C). The virus titers in AG129 mice infected with rHEP and rHEP^333^R were approximately 10^4^ or 10^2^ times higher than in S129 mice infected with rHEP and rHEP^333^R, respectively (Figure 4C). These results indicated that attenuated rHEP exhibits similar growth and virulence to the authentic pathogenic RABV in AG129 mice, intimating that the IFN responses may be related to the attenuation of rHEP in immunocompetent mice. Consistent with our observations on virus titration (Figure 4C), no significant difference in IFN gene expression levels was found between the two viruses in AG129 mice (Figure 4D). Interestingly, no significant difference was observed in expression levels of IFN-β gene or IFN-stimulated genes (ISGs) in S129 mice infected with rHEP or rHEP^333^R (Figures 4D–4F), despite the virus titer being markedly higher in rHEP^333^R-infected S129 mice (Figure 4C). Considering the relatively low virus titer and high IFN gene expression level of rHEP in S129 mice, rHEP may have induced IFN-β gene or ISGs expression more strongly than rHEP^333^R in S129 mice.

Immunohistochemistry of brain sections from rRABV-infected mice showed extensive viral infection in the neurons of AG129 mice infected with either rHEP or rHEP^333^R (Figures 4G and S3A). Notably, rHEP effectively infected astrocytes as well as neurons in AG129 mice compared with rHEP^333^R (Figures 4G, 4I, and S3B). In S129 mice infected with rHEP, a limited number of neurons and astrocytes were found to be positive for RABV N proteins (Figures 4H, S3C, and S3D). Even though rHEP^333^R showed RABV signals distributed across the whole brain in S129 mice, RABV-infected astrocytes were rarely observed in S129 mice (Figures 4H, S3C, and S3D). These results strengthen the hypothesis that astrocytes are highly susceptible to infection with rHEP but not rHEP^333^R *in vivo*.

### IFN induction and the sensitivity of rRABVs in neuronal cells

**Figure 5:**
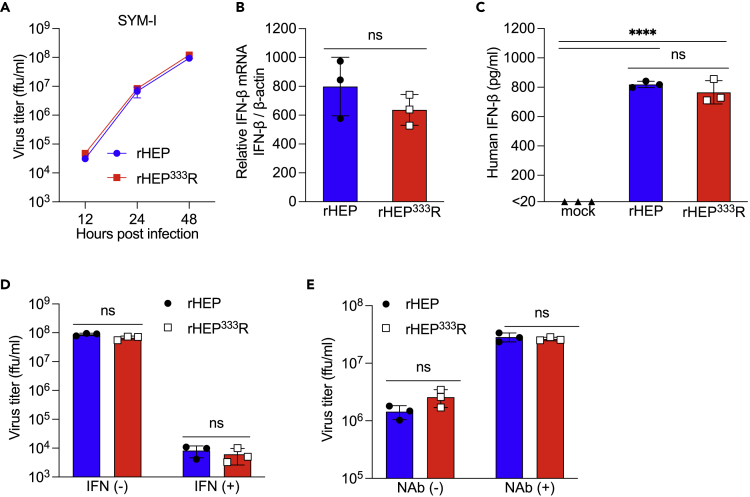
Susceptibility and resistance of RABV to IFN *in vitro* Human neuron-derived SYM-I cells were used for this series of experiments (A–C). SYM-I cells in a monolayer were inoculated with rHEP or rHEP^333^R at an MOI of 1. (A) Viral growth curve. Supernatants were collected at the indicated time points, and virus titers were measured by a focus forming assay. Means ± standard deviations of triplicate data from a representative experiment are shown in the graph. (B) IFN-β gene expression level quantified by qRT-PCR at 48 hpi. The data were normalized to the β-actin gene and presented as fold changes relative to the mock controls using the ΔΔCt method. (C) IFN-β protein level in culture supernatants was measured by ELISA at 48 hpi. (D) Stimulation IFN pathways by treatment with recombinant IFN-β. Cells were treated with exogenous human IFN-β at 600 ng/mL in culture medium for 16 h before RABV infection at an MOI of 0.1. Virus titers in the supernatants at 48 hpi was measured by a focus forming assay. (E) Neutralization of IFN signaling. Cells were treated with anti-human IFN receptor neutralizing antibody at 800 ng/mL in the culture medium for 16 h before RABV infection at an MOI of 0.1. Virus titer in the supernatant at 24 hpi was measured by a focus forming assay. All values in the graphs show the means ± standard deviations of triplicate data from a representative experiment. Statistical analysis was performed by multiple t tests using the Holm–Sidak method for (A), Student’s t test for (B), (D), and (E), and standard one-way ANOVA and Tukey’s multiple comparisons test for (C) (∗∗∗∗p < 0.0001, ns = not significantly different).

Finally, to further investigate the relationship between the IFN responses and pathogenicity of the rRABVs, we compared IFN inducibility and the sensitivity of rRABVs under an IFN-mediated antiviral state using a human neuroblastoma cell line SYM-I, in which IFN signaling pathways are validated (Honda et al., 1984). SYM-I cells were appropriate to evaluate the ability of IFN induction and the sensitivity of rRABVs because the growth kinetics of rRABVs were comparable in this cell line (Figure 5A). We then analyzed IFN-β responses against rRABV infection and found that IFN-β production was consistent between the two rRABVs both at the mRNA (Figure 5B) and the protein level (Figure 5C) in culture supernatants. These results indicated that there is no difference between rHEP and rHEP^333^R in terms of IFN-β inducibility in response to virus infection.

Next, the sensitivity of rRABVs to the antiviral state induced by IFN was examined. An antiviral state was induced in SYM-I cells by pretreatment with recombinant IFN-β before rRABV infection. Under these conditions, the proliferations of both rHEP and rHEP^333^R were inhibited in SYM-I cells (Figure 5D). By contrast, when the IFN signaling pathway was blocked by treatment of the cells with anti-IFN receptor neutralizing antibody, the progeny virus titer was increased at the same rate for rHEP and rHEP^333^R relative to the control infection (Figure 5E). These results suggested that rHEP and rHEP^333^R have similar potential to induce IFN and similar sensitivity to the subsequent antiviral state induced by IFN.

## Discussion

G protein plays major roles in RABV pathogenicity in terms of cell attachment, apoptosis induction, and stimulation of the host immune responses (Faber et al., 2002; Morimoto et al., 1999; Préhaud et al., 2003; Sarmento et al., 2005; Sasaki et al., 2018; Seif et al., 1985). In particular, an amino acid residue at position 333 in the G protein (G333) has been shown to be a major determinant of RABV pathogenicity and attenuation (Dietzschold et al., 1985; Tuffereau et al., 1989). In this study, to further understand the mechanism underlying the attenuation of RABV dependent on the amino acid residue at G333, we specifically focused on RABV tropism for astrocytes using an RABV nonpathogenic strain (rHEP) carrying Glu_333_ and a pathogenic mutant (rHEP^333^R) carrying Arg_333_ in G protein. We herein demonstrated that astrocytes are susceptible to infection with rHEP but not rHEP^333^R both *in vitro* and *in vivo*. These results indicated that the infectivity of the attenuated strain HEP in astrocytes is dependent on a single amino acid at G333 and that Glu_333_ has a higher affinity for astrocytes than Arg_333_.

Previous studies have reported that avirulent RABV strains including rHEP carrying Glu_333_ in G protein exhibit a broader cell tropism not specific to neuronal cells (Takayama-Ito et al., 2006; Tao et al., 2010) compared with pathogenic strains carrying Arg_333_ or Lys_333_. Our findings indicated that replacement of Arg_333_ to Glu_333_ increased astrocyte tropism of HEP strain. Even though the position G333 is not located at any of reported receptor binding sites on the G protein (Langevin et al., 2002; Lentz et al., 1982, 1984), replacement of Arg_333_ with Glu_333_ is known to greatly reduce the binding affinity of G protein to one of the RABV receptors, p75NTR (Tuffereau et al., 1998). Accordingly, the amino acid residue at G333 might influence the conformation of the receptor binding sites of G protein, possibly altering the RABV tropism for astrocytes.

In cells, virus infection induces IFN-β production immediately, and mass production of IFN-α/β is triggered by the earlier IFN-β response (Marié et al., 1998; Sato et al., 1998). Previously, abortive virus infection in astrocytes was found to be the main source of IFN production in the central nervous system (Pfefferkorn et al., 2016). Also, it has been reported that RABV infection of astrocytes is abortive (Pfefferkorn et al., 2016; Tian et al., 2018). In this study, viral RNA replication was detected in isolated astrocytes from RABV-infected S129 mice (Figures 3A and S3E); however, RABV N protein signals were rarely observed in astrocytes of S129 mice (Figures 4H and S3D) in contrast to strong signals in AG129 mice (Figures 4G and S3B). Based on these observations, infection of astrocytes in immunocompetent mice may be abortive with activated IFN responses as previously reported (Pfefferkorn et al., 2016; Tian et al., 2018). Besides, compared with rHEP, rHEP^333^R showed lower infectivity to astrocytes (Figures 1, 2, 3, and 4I) and consequently induced lower IFN-β production in astrocytes (Figures 2B, 3B, and 4D), which was independent of IFN inducibility or susceptibility to IFN (Figure 5). Yet, in the absence of an IFN response, rHEP showed an equivalent level of virus replication and pathogenicity to rHEP^333^R both *in vitro* (Figure 5E) and *in vivo* (Figures 4A–4C). These results suggested that IFN production in astrocytes is associated with attenuation of rHEP.

Previous studies have proposed several possible mechanisms of G333-dependent RABV attenuation. Even though HEP strain comprising Glu_333_ in G protein induces higher levels of apoptosis in infected neurons than pathogenic RABVs carrying Arg_333_ or Lys_333_ (Faber et al., 2002; Tao et al., 2010), another attenuated ERA strain carrying the Arg_333_ was reported to cause apoptosis (Morimoto et al., 1999). Moreover, it has been demonstrated that the poor neuronal transmissibility of HEP strain *in vivo*, which is one of the attenuation properties, was gained by the substitution of Glu_333_ to Arg_333_ in G protein. Conversely, a high pathogenic CVS strain lost its virulence through Arg_333_ mutation, still, it retained its distribution pattern in brain (Yan et al., 2002). Nonetheless, neuroinvasiveness was lost by an additional mutation at Lys_330_ in G protein (Coulon et al., 1998).

Although previous studies suggest that the amino acid at G333 contributes to the attenuation of RABV in multiple aspects in a strain-dependent manner indicated earlier, our novel proposed mechanism of Glu_333_-dependent astrocyte infection and IFN responses could partially account for the attenuation of HEP strain. Evidently, ERA strain comprising Arg_333_ in the G protein showed diminished infection of astrocytes (Potratz et al., 2020), which further supports the G333-dependent astrocyte infection. Controversially, recent studies have shown that other field RABV strains or even some bat-associated lyssaviruses could cause infection in astrocytes regardless of the amino acid at G333 or pathogenicity (Potratz et al., 2020; Klein et al., 2022). Based on these previous findings, we note that the amino acid at position 333 alone is not sufficient to determine astrocyte tropism of all RABV strains.

In conclusion, we have demonstrated here that rHEP infected astrocytes more efficiently than rHEP^333^R and consequently, evoked higher IFN responses in astrocytes *in vitro* and *in vivo*. In addition, we revealed that the IFN response is indispensable for both virulence and attenuation of rHEP using AG129 mice deficient in IFN responses. Taken together, our findings confirm that Glu_333_ in G protein is involved in the attenuation of rHEP, at least partially, by excessive infection of astrocytes triggering the induction of IFN. Our study provides new insights into the mechanism of RABV virulence attenuation and highlights the importance of astrocytes as potential drivers of the host immune response against lethal infection with RABV. In future studies, a profound understanding of RABV infection of astrocytes and the evoked immune responses should help to unravel the mechanism of RABV attenuation.

### Limitations of the study

Our results suggested that the IFN-mediated antiviral activity in astrocytes could, at least in part, lead to the attenuation of RABV pathogenicity. However, we cannot exclude the possibility that neuronal cells, as well as astrocytes, also induce an IFN-mediated antiviral responses and are involved in the attenuation of RABV pathogenicity; this is because inhibition of the IFN pathway generally improves the growth of various viruses. Another limitation is the choice of the RABV strains. In our study, we used a laboratory fixed strain HEP-Flury, but infectivity of astrocytes is variable among RABV strains (Potratz et al., 2020). Thus, comparison using different RABV strains including RABV street strains will provide a better understanding of inter-strain variations. In addition, further investigations are needed to elucidate the specific mechanism of clearance of RABVs dependent on the amino acid at G333 that results in reduced pathogenicity.

## STAR★Methods

### Key resources table

**Table:** 

REAGENT or RESOURCE	SOURCE	IDENTIFIER
**Antibodies**		

Anti-human IFN-receptor antibody (mouse) [MAR1-5A3]	Santa Cruz	Cat# sc-53591; RRID: AB_783928
Anti-NeuN antibody (mouse) [ab104224]	Abcam	Cat# ab104224; RRID: AB_10711040
Anti-GFAP antibody (rabbit) [G9269]	Sigma Aldrich	Cat# G9269; RRID: AB_477035
Anti-RABV N antibody (mouse) [3R7-5B12]	HyTest	Cat# 3R7-5B12; RRID: AB_1621793
Alexa Fluor 488-anti-mouse IgG2a antibody (goat) [A-21131]	Invitrogen	Cat# A21131; RRID: AB_141618
Alexa Fluor Plus 594-anti-rabbit IgG (H+L) antibody (goat) [A-32740]	Invitrogen	Cat# A32740; RRID: AB_2762824
Alexa Fluor 647-anti-mouse IgG2b antibody (goat) [A-21242]	Invitrogen	Cat# A21242; RRID: AB_1500900
APC-anti-mouse ACSA2 antibody (human) [130-116-245]	Miltenyi Biotec	Cat# 130-116-245; RRID: AB_2727423
Alexa Fluor 488-anti-mouse CD11b antibody (rat) [53-0112-82]	Invitrogen	Cat# 53-0112-82; RRID: AB_469901
FITC-anti-RABV N antibody (mouse) [806001]	FUJIREBIO	Cat# 800-092; RRID: AB_2802166

**Bacterial and virus strains**		

Rabies virus (HEP-Flury)	Dr. Chang-Kweng, National Institute of Infectious Diseases	GenBank: AB085828.1
Recombinant Rabies virus (rHEP)	This study	N/A
Recombinant Rabies virus (rHEP^333^R)	This study	N/A

**Chemicals, peptides, and recombinant proteins**		

Hoechst 33342, Trihydrochloride, Trihydrate - FluoroPure Grade	Molecular Probes	Cat# H21492
Recombinant human IFN-β	This paper	N/A

**Critical commercial assays**		

One Step TB Green PrimeScript PLUS RT-PCR Kit	Takara Bio	Cat# PR096A
THUNDERBIRD Probe One-step qRT–PCR kit	TOYOBO	Cat# QRZ-101
Mouse IFN-beta ELISA Kit	R&D	Cat# 42400-1
Adult Brain Dissociation Kit	Miltenyi Biotec	Cat# 130-107-677
Expi293 Expression System Kit	Gibco	Cat# A14635

**Experimental models: Cell lines**		

Mouse: NA cells	Dr. F. A. McMorris, The Wistar Institute	N/A
Human: SYM-I cells	Dr. A. Kawai, Research Institute for Production and Development	N/A
Hamster: BHK/T7-9 cells	Riken BioResource Research Center	# RCB4942
Human: SVG-A cells	Dr. W. J. Atwood, Brown University	N/A
Human: Expi293F cells	Gibco	Cat# A14635
Mouse: Primary astrocytes	This study	N/A

**Experimental models: Organisms/strains**		

Mouse: S129 (129S7/SvEvBrdBkl-Hprt^b-m2^)	Marshall BioResources	N/A
Mouse: AG129 (IFNα/β/γR^−/−^)	Marshall BioResources	N/A

**Oligonucleotides**		

Forward qPCR primer for RABV HEP N: 5'-GCCACGGTTATTGCTGCAT-3′	This paper	N/A
Reverse qPCR primer for RABV HEP N: 5'-CTCCCAAATAGCCCCCTAGAA-3′	This paper	N/A
TaqMan probe for RABV HEP N: 5'- FAM-CCC TCA TGA GAT GTC-MGB -3′	This paper	N/A
Forward qPCR primer for mouse IFN-β: 5'-ATGAGTGGTGGTTGCAGGC-3′	Kamizaki et al. (2004)	N/A
Reverse qPCR primer for mouse IFN-β: 5'-TGACCTTTCAAATGCAGTAGATTCA-3′	Kamizaki et al. (2004)	N/A
TaqMan probe for mouse IFN-β: 5'- FAM-AAG CAT CAG AGG CGG ACT CTG GGA-TAMRA -3′	Kamizaki et al. (2004)	N/A
Forward qPCR primer for mouse Mx1: 5'-CAATGATCCTTTAGCTGCTAACCTTA-3′	Deonarain et al. (2004)	N/A
Reverse qPCR primer for mouse Mx1: 5'-GTTTACAAAGGGCTTGCTTGCT-3′	Deonarain et al. (2004)	N/A
TaqMan probe for mouse Mx1: 5'- FAM-TCA GAA TGT TGC CTT TAG ACT GTG G-TAMRA -3′	Deonarain et al. (2004)	N/A
Forward qPCR primer for mouse OAS: 5'-TGAGCGCCCCCCATCT-3′	Deonarain et al. (2004)	N/A
Reverse qPCR primer for mouse OAS: 5'-CATGACCCAGGACATCAAAGG-3′	Deonarain et al. (2004)	N/A
TaqMan probe for mouse OAS: 5'- FAM-AGG AGG TGG AGT TTG ATG TGC TG-TAMRA -3′	Deonarain et al. (2004)	N/A
Forward qPCR primer for mouse IFIT2: 5'-GGGAAAGCAGAGGAAATCAA-3′	Davis et al. (2017)	N/A
Reverse qPCR primer for mouse IFIT2: 5'-TGAAAGTTGCCATACAGAAG-3′	Davis et al. (2017)	N/A
TaqMan probe for mouse IFIT2: 5'- FAM-ATG CGT CCT TAG TCG GCT TTC TC-TAMRA -3′	Davis et al. (2017)	N/A
Forward qPCR primer for human IFN-β: 5'-AAACTCATGAGCAGTCTGCA-3′	Jaworska et al. (2007)	N/A
Reverse qPCR primer for human IFN-β: 5'-AGGAGATCTTCAGTTTCGGAGG-3′	Jaworska et al. (2007)	N/A
TaqMan probe for human IFN-β: 5'- FAM-ATG GTC CAG GCA CAG TGA CTG TCC TC-BHQ1 -3′	Jaworska et al. (2007)	N/A
human Mx1	Integrated DNA Technologies	Cat# Hs.PT.58.40261042
Pre-Developed TaqMan Assay Reagent Mouse ACTB	Applied Biosystems	Cat# 4352933E
Pre-Developed TaqMan Assay Reagent Human ACTB	Applied Biosystems	Cat# 4352935E
Forward qPCR primer for mouse MAP2: 5'-AGACCTTCCTCCATCCTCCC-3′	Li et al. (2018)	N/A
Reverse qPCR primer for mouse MAP2: 5'-GCCACTTTTTCCTGCTCTGC-3′	Li et al. (2018)	N/A
Forward qPCR primer for mouse Iba1: 5'-CTTGAAGCGAATGCTGGAGAA-3′	Carrano and Das (2015)	N/A
Reverse qPCR primer for mouse Iba1: 5'-GCAGCTCGGAGATAGCTTT-3′	Carrano and Das (2015)	N/A
Forward qPCR primer for mouse GFAP: 5'-GCTGGAGGGCGAAGAAAACCG-3′	Zhang et al. (2019)	N/A
Reverse qPCR primer for mouse GFAP: 5'-CACGGATTTGGTGTCCAGGCTGG-3′	Zhang et al. (2019)	N/A

**Recombinant DNA**		

pHEP	This study	N/A
pHEP^333^R	This study	N/A
pCXSN-hIFN-β-his	This study	N/A

**Software and algorithms**		

GraphPad Prism version 9.2.0	GraphPad Software	https://www.mdf-soft.com
QuantStudio 7 Flex Real-Time PCR System	Applied Biosystems	https://www.thermofisher.com
QuantStudio 7 Flex Real-Time PCR System Software	Applied Biosystems	https://www.thermofisher.com
Fluorescence microscopy, IX73	Olympus	https://www.olympus-lifescience.com
Zeiss LSM 780	Zeiss	https://www.zeiss.co.jp/microscopy/products/confocal-microscopes.html
Zeiss ZEN software	BD Biosciences	https://www.zeiss.co.jp/microscopy/downloads.html
BD FACSMelody Cell Sorter	BD Biosciences	https://www.bdbiosciences.com/ja-jp/products/instrumeants/flow-cytometers/research-cell-sorters/bd-facsmelody
BD FACSChorus software	BD Biosciences	https://www.bdbiosciences.com/en-in/products/software/instrument-software/bd-facschorus-software

### Resource availability

#### Lead contact

Further information and requests for resources should be directed to and will be fulfilled by the lead contact, Michihito Sasaki (m-sasaki@czc.hokudai.ac.jp).

#### Materials availability

Plasmid constructs generated in this study will be available upon request with a material transfer agreement (MTA).

### Experimental model and subject details

#### Cell lines

Mouse neuroblastoma (NA) cells, human neuroblastoma (SYM-I) cells and baby hamster kidney cells stably expressing T7 RNA polymerase (BHK/T7-9) (Ito et al., 2001) were maintained in Eagle’s Minimum Essential Medium supplemented with 10% fetal bovine serum (FBS). Simian virus 40 (SV40)-transformed human fetal astrocyte (SVG-A) cells were propagated in Dulbecco’s Modified Eagle’s Medium (DMEM) supplemented with 10% FBS. SYM-I cells were cultured in type-I collagen-coated plates. All the cells above were incubated at 37 °C in the presence of 5% CO_2_. Expi293F cells derived from the human 293 cell line were maintained in Expi293 Expression Medium (Gibco) in spinner flasks at 37 °C in the presence of 8% CO_2_.

#### Primary cell culture

Primary cultured astrocytes were prepared from S129 mice. The whole brains from 4–5-day-old mouse pups were minced, and then incubated with papain (10 U/mL) and DNase (0.1 mg/mL) for 20 min. Dissociated cells were suspended in DMEM/Ham’s F-12 containing 10% FBS, 100 U/mL penicillin and 0.1 mg/mL streptomycin. The cell suspension was seeded into a poly-*l*-lysine-coated T25 flask. After 7–8 days, the flask was shaken at 250 rpm at 37 °C for at least 12 h to remove all cells except astrocytes. Adherent cells were detached with trypsin and re-seeded onto poly-*l*-lysine-coated 24-well plates at a density of 8.0 × 10^3^ cells/cm^2^ for virus inoculation.

#### Animals

Immunocompetent S129 mice and type-I/II IFN receptor-KO AG129 mice (in an S129 background) were obtained from Marshall BioResources. S129 and AG129 mice were maintained in our laboratory. Sex-matched 12-weeks-old S129 and AG129 mice were used for the animal experiments. Animal experiments were approved by the Institutional Animal Care and Use Committee of Hokkaido University (approval number 19-0014) and were performed in accordance with the committee’s guidelines.

#### Viruses

Recombinant clones of RABV (rRABV) were used in this study. The RABV HEP-Flury strain, kindly provided by Dr. Chang-Kweng Lim (National Institute of Infectious Diseases, Japan), was used as a template to generate a plasmid vector carrying the full-length cDNA of HEP strain (pHEP), as previously described (Anindita et al., 2016). The Q333R mutation was introduced into the G gene by PCR-based mutagenesis and the fragment carrying the Q333R mutation was subcloned into the pHEP plasmid (pHEP^333^R). Virus recovery was performed following a previously described procedure with some modifications (Anindita et al., 2016; Ito et al., 2001). Briefly, BHK/T7-9 cells were co-transfected with pHEP or pHEP^333^R, and helper plasmids pT7IRES-RN, -RP and -RL. At 5 days post-transfection, the culture supernatants were passaged in NA cells for virus propagation. The infectious virus titers of rHEP and rHEP^333^R were determined by a focus forming assay performed as previously described (Anidita et al., 2016).

### Methods details

#### Quantitative real-time RT-PCR

The mRNA or viral RNA copy numbers were quantified with the Thunderbird Probe One-step qRT-PCR Kit (TOYOBO) and TaqMan probe/primer sets specifically targeting RABV: HEP N (F: 5ʹ-GCC ACG GTT ATT GCT GCA T-3ʹ, R: 5ʹ-CTC CCA AAT AGC CCC CTA GAA-3ʹ, Probe: 5ʹ-FAM-CCC TCA TGA GAT GTC-MGB-3ʹ), mouse IFN-β (F: 5ʹ-ATG AGT GGT GGT TGC AGG C-3ʹ, R: 5ʹ-TGA CCT TTC AAA TGC AGT AGA TTC A-3ʹ, Probe: 5ʹ-FAM-AAG CAT CAG AGG CGG ACT CTG GGA-TAMRA-3ʹ), mouse Mx1 (F: 5ʹ-CAA TGA TCC TTT AGC TGC TAA CCT TA-3ʹ, R: 5ʹ-GTT TAC AAA GGG CTT GCT TGC T-3ʹ, Probe: 5ʹ-FAM-TCA GAA TGT TGC CTT TAG ACT GTG G-TAMRA-3ʹ), mouse OAS (F: 5ʹ-TGA GCG CCC CCC ATC T-3ʹ, R: 5ʹ-CAT GAC CCA GGA CAT CAA AGG-3ʹ, Probe: 5ʹ-FAM-AGG AGG TGG AGT TTG ATG TGC TG-TAMRA-3ʹ), mouse IFIT2 (F: 5ʹ-GGG AAA GCA GAG GAA ATC AA-3ʹ, R: 5ʹ-TGA AAG TTG CCA TAC AGA AG-3ʹ, Probe: 5ʹ-FAM-ATG CGT CCT TAG TCG GCT TTC TC-TAMRA-3ʹ), human IFN-β (F: 5ʹ-AAA CTC ATG AGC AGT CTG CA-3ʹ, R: 5ʹ-AGG AGA TCT TCA GTT TCG GAG G-3ʹ, Probe: 5ʹ-FAM-ATG GTC CAG GCA CAG TGA CTG TCC TC-BHQ1-3ʹ). A predesigned qPCR assay was purchased for human Mx1 (Hs.PT.58.40261042) from Integrated DNA Technologies. The expression levels of house-keeping genes were quantified using Pre-Developed TaqMan Assay Reagent Mouse ACTB or Pre-Developed TaqMan Assay Reagent Human ACTB (Applied Biosystems). The One Step TB Green PrimeScript PLUS RT-PCR Kit (Takara Bio) was used to determine the mRNA copy number along with primer sets specifically targeting: mouse MAP2 (F: 5ʹ-AGA CCT TCC TCC ATC CTC CC-3ʹ, R: 5ʹ-GCC ACT TTT TCC TGC TCT GC-3ʹ), mouse Iba1 (F: 5ʹ-CTT GAA GCG AAT GCT GGA GAA-3ʹ, R: 5ʹ-GCA GCT CGG AGA TAG CTT T-3ʹ) and mouse GFAP (F: 5ʹ-GCT GGA GGG CGA AGA AAA CCG-3ʹ, R: 5ʹ-CAC GGA TTT GGT GTC CAG GCT GG-3ʹ).

#### Recombinant human IFN-β

The cDNA fragment of human IFN-β was obtained from the transcripts of SYM-I cells infected with RABV by RT-PCR and cloned into pCXSN vector fused with a 6×-histidine tag (pCXSN-hIFN-β-his). Recombinant human IFN-β was expressed using the Expi293 Expression System (Gibco) following the manufacturer’s procedure. Briefly, pCXSN-hIFN-β-his (30 μg) was transfected by ExpiFectamine 293 Reagent into Expi293F cells prepared at 3 × 10^6^ cells/mL in 30 mL of suspension culture. Twenty hours post-transfection, the provided enhancers were added to the flask and the cell culture supernatant was harvested at 6 days post-transfection. Recombinant human IFN-β in the supernatant was purified using Ni Sepharose Excel (Cytiva) following the manufacturer’s protocols, with 400 mM imidazole for elution. The recovery and bioactivity of human IFN-β were confirmed by immunoblotting and quantification of the mRNA of the IFN-stimulated gene *in vitro*, respectively.

#### Quantification of IFN, and inhibition or stimulation of the IFN pathway in rRABV-infected cells

SYM-I cells cultured in collagen-coated 24-well plates were infected with rRABV at a multiplicity of infection (MOI) of 1. The expression levels of mRNA and protein of IFN-β were quantified by qRT-PCR and a Mouse IFN-beta ELISA Kit (R&D), respectively.

To inhibit IFN stimulation, SYM-I cells were treated with anti-human IFN-receptor antibody (MAR1-5A3, Santa Cruz) at 800 ng/mL for 16 h. Then, cells were infected with rRABVs at an MOI of 0.1 and the virus titer in the cell culture supernatant was determined at 24 h post-infection (hpi).

To stimulate IFN signaling, recombinant human IFN-β was added to the SYM-I cell culture at 600 ng/mL followed by 16 h of incubation. rRABVs were used to infect the cells at an MOI of 0.1 and culture supernatants were collected for virus titration at 48 hpi.

#### Animal experiments

Sex-matched 12-weeks-old S129 and AG129 mice were used for our animal studies. Mice were intracranially inoculated with 20 μL of PBS with or without 10^4^ focus forming units (ffu) of rRABV under anesthesia. All mice in the survival groups were observed for 13 days for signs of symptoms and bodyweight changes. The humane endpoint was defined as a 20% decrease in body weight or an inability to reach food or water because of the disease onset. For further analysis, mice were euthanized, and brain tissues were harvested at 5 days post-infection (dpi). A suspension containing 10% brain homogenate in PBS was used for virus titration and RNA extraction.

#### Immunohistochemistry

Brain tissues harvested from rRABV-infected mice at 5 dpi were fixed in 10% phosphate-buffered formalin for more than 48 h and then embedded into paraffin blocks. The paraffin blocks were sectioned at a 4-μm thickness and mounted on Platinum PRO micro glass slides (Matsunami). The sections on slides were subjected to antigen retrieval in citrate buffer for 5 min by a pressure cooker. The slides were then treated with 10% goat serum (Nichirei Biosciences) for 1 h at room temperature and incubated at 4 °C overnight with primary antibodies against the following proteins: NeuN (ab104224; Abcam, 1:1000), GFAP (G9269; Sigma Aldrich, 1:400) or RABV N protein (3R7-5B12; Hytest, 1:100). After three-times washes with PBST (0.01% Tween 20 in PBS), secondary staining was performed with Alexa Fluor 488-anti-mouse IgG2a antibody (A-21131; Invitrogen, 1:1000), Alexa Fluor Plus 594-anti-rabbit IgG (H+L) antibody (A32740; Invitrogen, 1:1000) and Alexa Fluor 647-anti-mouse IgG2b antibody (A-21242; Invitrogen, 1:1000) in the presence of 10 μg/mL Hoechst 33,342 for 1 h at room temperature. Microscopic analysis was conducted by LSM780 and ZEN software (Zeiss).

#### Isolation of astrocytes from mouse brains

Mouse brains were dissociated to cells using the Adult Brain Dissociation Kit (Miltenyi Biotec) following the manufacturer’s protocol with some modifications. Briefly, a fresh whole brain was washed with cold PBS and dissected into small pieces. Aliquots were transferred into C Tubes (Miltenyi Biotec) and mixed with the provided enzymes. C Tubes were attached to the gentleMACS Dissociator (Miltenyi Biotec), and the following gentleMACS programs were performed: m_brain_01_01, m_brain_02_01, m_brain_03_01. Each program was repeated twice with 5-min intervals on a tube rotator at 37 °C. The dissociated brain was applied onto a 70 μm cell strainer and debris and red cell removal steps were performed in accordance with the manufacturer’s protocol.

The separated cells were treated with 1% bovine serum albumin (BSA) in PBS for 30 min, followed by immunostaining with APC-anti-mouse ACSA2 antibody (130-116-245; Miltenyi Biotec, 1:50) and Alexa Fluor 488-anti-mouse CD11b antibody (53-0112-82; Invitrogen, 1:600) for 30 min at 4 °C in the dark. After three-times washes with 0.1% BSA in PBS, cells were separated by BD FACSMelody Cell Sorter along with BD FACSChorus software (BD Biosciences).

### Quantification and statistical analysis

All statistical analyses were performed using GraphPad Prism software. For analyses between two groups, a two-tailed unpaired Student’s *t*-test was used. For the comparison of two groups at multiple time points, a multiple *t*-test by the Holm–Sidak method was performed. For comparisons among more than two groups, one-way ANOVA with Tukey’s multiple comparisons test was used (∗p < 0.05, ∗∗p < 0.01, ∗∗∗∗p<0.0001, ns=not significantly different). Data were presented as the mean ± standard deviation (SD) in graphs.

## Data Availability

All data reported in this paper will be shared by the lead contact upon request. This paper does not report original code. Any additional information required to reanalyze the data reported in this paper is available from the lead contact upon request.
