# Supplementary material for: Glu333 in rabies virus glycoprotein is involved in virus attenuation through astrocyte infection and interferon responses
Source: iScience. 2022 Mar 22;25(4):104122. doi: 10.1016/j.isci.2022.104122 (PMC8983343; doi:10.1016/j.isci.2022.104122)
Supplement: Document S1. Figures S1–S4 [file mmc1.pdf]

iScience, Volume *25*

Supplemental information

Glu_{333} in rabies virus glycoprotein is involved in virus attenuation through astrocyte infection and interferon responses

Yukari Itakura, Koshiro Tabata, Kohei Morimoto, Naoto Ito, Herman M. Chambaro, Ryota Eguchi, Ken-ichi Otsuguro, William W. Hall, Yasuko Orba, Hirofumi Sawa, and Michihito Sasaki

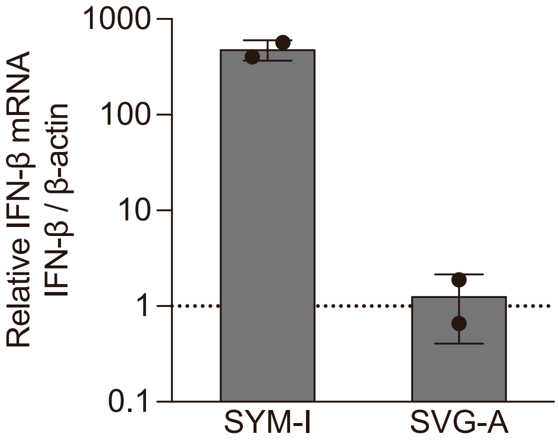

Fig. S1. Expression of IFN-β mRNA of neuron-derived SYM-I cell and astrocyte-derived SVG-A cell in response to RABV infection. Related to Figure 2.

SYM-I cell and SVG-A cell in a monolayer were inoculated with rHEP at an MOI of 1. IFN-β gene expression level quantified by qRT-PCR at 24 hpi. The data were normalized to the β-actin gene and presented as fold changes relative to the mock controls using the ΔΔCt method. The graphs show the means ± standard deviations of duplicate data.

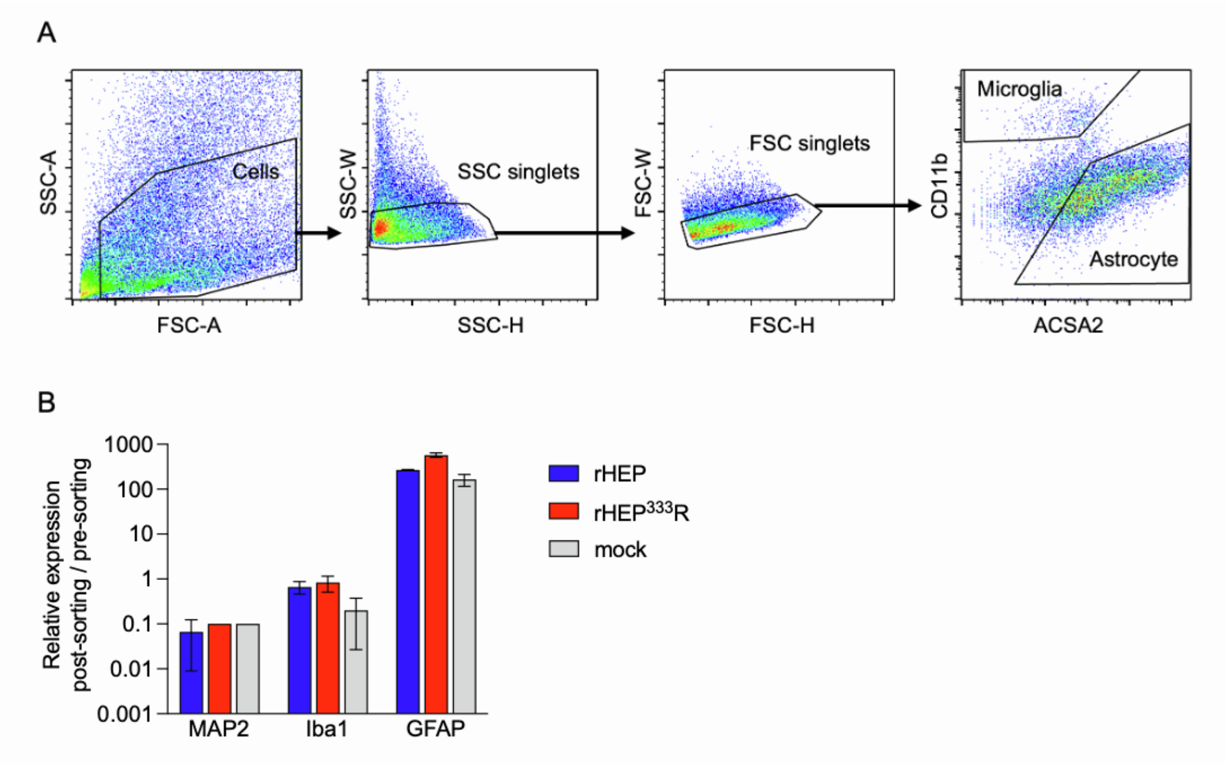

Fig. S2. Isolation of astrocytes from mouse brains. Related to Figure 3.

(A) Gating strategy of astrocytes from mouse brains. Mouse brains were dissociated with the Adult Brain Dissociation Kit (Miltenyi Biotec) and dissociated cells were labeled with antibodies against CD11b for microglia and ACSA2 for astrocytes. Cell populations were gated as singlets based on the profiles of SSC and FSC. According to the cytogram of CD11b and ACSA2, ACSA2-positive astrocytes were sorted by the FACSMelody Cell Sorter along with FACSChorus software (BD Biosciences). Images were visualized by FlowJo (BD Biosciences). (B) Isolation of astrocytes from mouse brains was confirmed by examination of the mRNA expression of cell markers: MAP2 for neurons, Iba1 for microglia and GFAP for astrocytes. The data were normalized to the β-actin gene and presented as fold changes relative to the dissociated whole brain prior to the astrocyte isolation using the ΔΔCt method. High expression levels of the astrocyte marker (GFAP) were observed for the sorted cells, whereas the expression levels of the neuron marker (MAP2) and microglia marker (Iba1) were decreased compared with those of the pre-sorted cells.

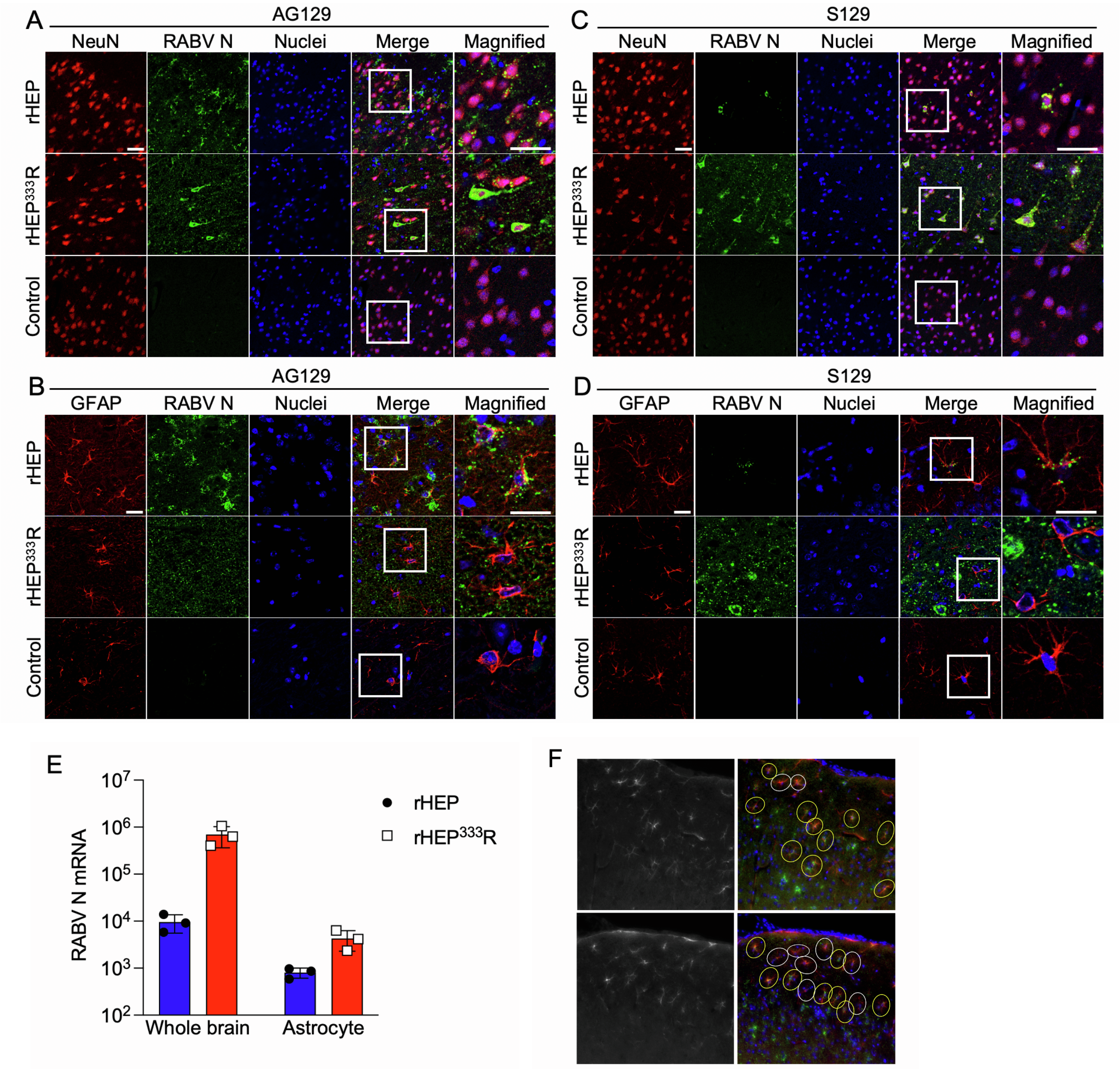

G

	Cerebral cortex			Hippocampus		
	Counted cell number	RABV positive %	SD	Counted cell number	RABV positive %	SD
rHEP	93	69.04	5.02	149	76.23	10.75
$rHEP^{333}R$	92	29.31	12.29	121	32.05	15.44

Fig. S3. RABV infection in IFN-receptor knockout mice. Related to Figure 4.

Twelve-week-old AG129 or S129 mice were intracranially inoculated with 10^4 ffu of rHEP or $rHEP^{333}R$. (A–D) Brain sections of AG129 and S129 mice at 5 dpi were stained for (A, C) NeuN or (B, D) GFAP, and RABV N protein. Scale bar; 20 µm. (E) RABV N mRNA copies in mouse brain and isolated astrocytes at 5dpi. The data were quantified by the standard curve method and normalized to the β-actin gene expression. (F) Examples of counting astrocytes. White circle; RABV-negative, yellow circle; RABV-positive. (G) Summary of counting RABV-positive astrocytes in cerebral cortex and hippocampus of AG129 mice.

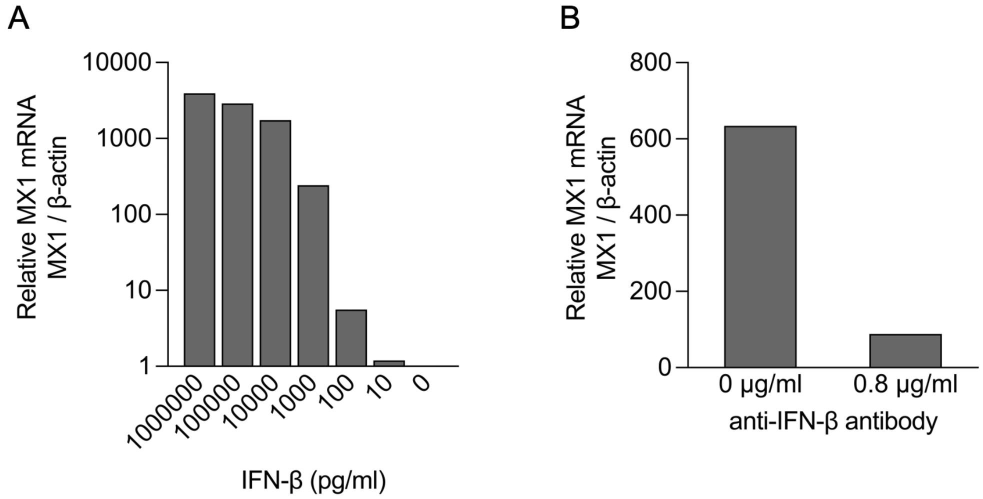

Fig. S4. Stimulation and inhibition of IFN signaling. Related to Figure 5.

Human neuroblastoma cell line SYM-I was used for the experiments. (A) Stimulation of IFN signaling by treatment with recombinant IFN-β. Cells were treated with exogenous human IFN-β at the indicated concentration for 16 hours at 37°C. Thereafter, RNA was extracted from the cells and subjected to qRT-PCR of the Mx1 gene, an IFN-stimulated gene. The data were normalized to the β-actin gene and presented as fold changes relative to the mock controls using the ΔΔCt method. (B) Neutralization of IFN signaling. Cells were treated with anti-human IFN receptor neutralizing antibody at 800 ng/ml in the culture medium for 16 hours prior to rRABV infection at an MOI of 0.1. RNA was extracted at 24 hours post-infection (hpi) and subjected to qRT-PCR of the Mx1 gene. The data were normalized to the β-actin gene and presented as fold changes relative to the controls without the antibody using the ΔΔCt method. All values in the bar graphs show the means of duplicates of a representative experiment.
